# White-Tailed Eagles’ (*Haliaeetus albicilla*) Exposure to Anticoagulant Rodenticides and Causes of Poisoning in Poland (2018–2020)

**DOI:** 10.3390/toxics10020063

**Published:** 2022-02-01

**Authors:** Bartosz Sell, Tomasz Śniegocki, Marta Giergiel, Andrzej Posyniak

**Affiliations:** Department of Pharmacology and Toxicology, National Veterinary Research Institute, Partyzantow 57, 24-100 Pulawy, Poland; sniego@piwet.pulawy.pl (T.Ś.); marta.giergiel@piwet.pulawy.pl (M.G.); aposyn@piwet.pulawy.pl (A.P.)

**Keywords:** anticoagulant rodenticides, poisoning, Poland, birds of prey, white-tailed eagle

## Abstract

The white-tailed eagle (*Haliaeetus albicilla*) is strictly protected in Poland due to its threat of extinction. This study’s main goal was to assess their exposure to indirect poisoning by anticoagulant rodenticides (AR). This study presents the investigation results of 40 white-tailed eagles’ suspected poisoning cases in the years 2018–2020 in Poland. In all tested liver samples, using a liquid chromatography–mass spectrometry method, at least one of the AR (bromadiolone, brodifacoum, difenacoum, flocoumafen) was detected and confirmed. The other tested AR compounds (chlorophacinone, coumachlor, coumatetralyl, difethialone, diphacinone, warfarin) were not detected. The mean concentration of the sum of rodenticides was 174.4 µg/kg (from 2.5 to 1225.0 µg/kg). In 20 cases, the sum concentration was above 100 µg/kg and in 10 cases it was above 200 µg/kg. Interpretation of cases of AR poisonings should take into account their concentration in the liver, anatomopathological lesions, circumstances of death/finding of the animal, and elimination of other possible causes of poisoning. Based on this study, AR was the direct cause of death in 10% of incidents. Extensive use of rodenticides generates a high risk of poisonings of white-tailed eagles in Poland.

## 1. Introduction

The discovery of anticoagulant rodenticides and their high efficacy significantly changed the game in the field of rodent control and led to their widespread and extensive use worldwide [1]. Their mechanism of action (inhibiting the activation of vitamin K) caused clotting disorders and gradual bleeding of animals [2,3]. The advantage of their application was a delayed toxic effect in relation to the time of poison ingestion, which eliminated the problem of learned aversion and caution of rodents regarding the introduction of new food observed with previously used poisons. First-generation anticoagulant rodenticides (FGARs) (e.g., warfarin, coumachlor) had a relatively short elimination time from the body [4]. They required several exposures to induce a toxic effect, and their extensive use tended to lead to occurrences of genetic resistance [1,5,6]. In the 1970s, the discovery of second-generation anticoagulant rodenticides (SGARs) (e.g., bromadiolone, brodifacoum) resulted in a significant increase in their efficacy [3,4,7,8]. Their potential to bioaccumulate in the body (especially in liver tissue) resulted in a longer duration of toxic action [4,9,10]. In many cases, a single ingestion of poison is sufficient to kill the rodents [1,3,4,6,7,8,10]. At the same time, products containing SGARs still have the character of compounds with delayed action. Unfortunately, with the increase in effectiveness, the risk of unintentionally poisoning domestic and wild animals has increased, both directly through the ingestion of the poison and indirectly by ingesting poisoned animals—especially considering the increased possibility of prey being hunted when already weakened by the poison [1,3,4,6,11,12,13]. There were observed behavioral changes in birds of prey, notable changes in hunting areas caused by extensive use of rodenticides in specific regions [12]. In contrast to Walter et al. [14], who suggested that the risk of indirect poisoning by brodifacoum (one of the SGARs) is minimal, we found many publications documenting cases of direct and indirect AR poisonings of wildlife [1,4,5,8,13,15,16,17,18,19]. This was based on the fact that none of the 70 labeled Norway rats was caught or removed by wild predators or scavengers [14]. The SGARs have a tendency to persist in organs containing vitamin K epoxide reductase, such as the liver, kidney, or pancreas [20]. Taking this and the available literature into account in most cases, the liver is the dedicated tissue for AR analysis [1,9,10,21,22,23]. In some European countries, there are organized programs to assess the occurrence of human and animal health exposures to different types of xenobiotics, including AR [19,24]. Gomez et al. [19] also pointed out the lack of data from Eastern European regions. For many years, carbofuran or other carbamate pesticides, such as aldicarb or bendiocarb, have been the most detected analytes in cases of fatal wildlife poisoning [25,26,27,28]. However, in addition to these, ARs are often also detected in birds of prey.

This research allows us to estimate the prevalence of, and assess exposure to, AR poisonings of white-tailed eagles (*Haliaeetus albicilla*) in Poland based on analyses of test results of 40 liver samples. For toxicological investigations, we used a previously published method that allows the analysis of compounds from the groups belonging to rodenticides, carbamate, and organophosphate pesticides, coccidiostats and mycotoxins [29].

The white-tailed eagle (*Haliaeetus albicilla*) is the largest predatory and scavenging free-living species of bird in Poland, also generally widely distributed across Eurasia [30,31,32]. The body mass of adult individuals reaches 4 to 5 kg, and their wingspan up to 2.5 m [32]. In recent decades, the population of the white-tailed eagle in Poland has shown a growing trend, which also results in a larger area of occurrence. It is most likely to nest in older forests on large trees, near feeding grounds that are rich in fish and waterbirds [30,32]. The diet of the white-tailed eagle is mainly fish, but also includes animals such as birds, mammals (including red fox *Vulpes vulpes*), and occasionally carrion [30,33]. This is the first report about white-tailed eagles’ (*Haliaeetus albicilla*) exposure to AR in Poland.

## 2. Materials and Methods

### 2.1. Samples Collection

**Figure 1:**
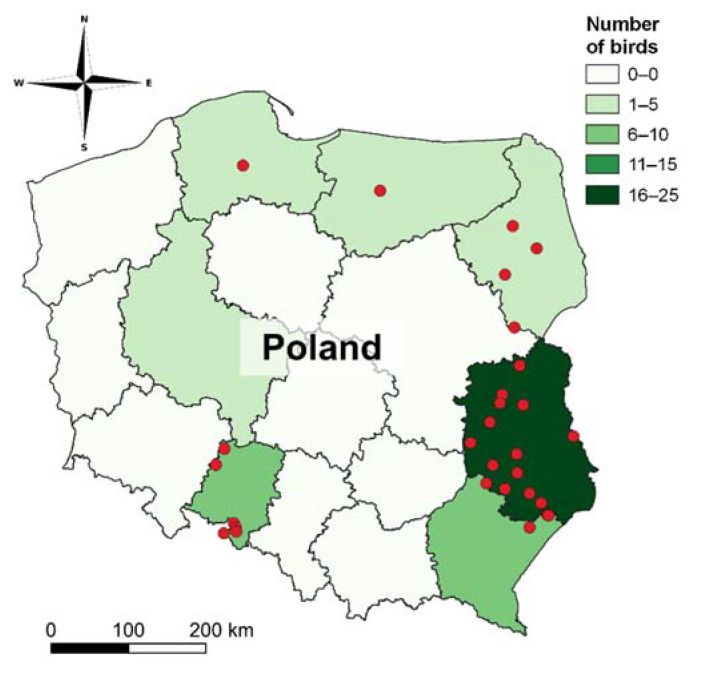
Sampling locations of investigated white-tiled eagles (map of sampling sites was created using QGIS software version 3.22 (QGIS.org, 2022)).

The samples were collected from dead birds across different regions of Poland, mostly from the eastern province (Figure 1), and delivered to the laboratory. Most samples were collected in the winter and delivered in a frozen state. Ten birds were fully autopsied and a further twenty were observed during sampling. In six cases, the assessment was difficult due to the partial degree of decomposition of the material provided. Some of our samples were found even several weeks after death (based on the presence of indicator parasites). Sometimes, the observation of all anatomopathological lesions was also complicated by the previously frozen state of the samples. Additional information that we were able to collect regarding anatomopathological lesions and observations of the samples received, as well as age and sex of the birds, are presented in Table S1. Samples were collected by Forest Inspectorates, Provincial Inspectorates of Environmental Protection, and private citizens. Liver samples were secured before analysis, due to the AR’s predisposition to accumulate in it. After sampling, the liver samples were stored in a freezer below −18 °C.

### 2.2. Analytical Method

First, the samples were scanned according to the scope of our previously described method [29], which allows detection of rodenticides (bromadiolone, brodifacoum, chlorophacinone, coumachlor, coumatetralyl, difenacoum, diphacinone, flocoumafen, warfarin, strychnine), carbamate pesticides (aldicarb, bendiocarb, carbaryl, carbofuran, dioxacarb, propoxur), organophosphorus pesticides (azinphos-ethyl, azinphos-methyl, bromophos-ethyl, carbophenothion, chlorfenvinphos, chlorpyrifos, diazinon, dichlofenthion, dicrotophos, dimefox, dimethoate, disulfoton, ethion, etrimfos, fonofos, malaoxon, malathion, methacrifos, methamidophos, mevinphos, omethoate, paraoxon, parathion-ethyl, parathion-methyl, phosalone, pirimiphos-ethyl, pirimiphos-methyl, propetamphos, pyrazophos, sulfotep), coccidiostats (lazalocid, maduramycin, monensin, narasin, salinomycin, semduramycin), and micotoxins (aflatoxin B1, aflatoxin B2, aflatoxin G1, aflatoxin G2, deoxynivalenol, sterygmotocystin, toxin HT-2, toxin T-2, zearalenone). Then, the list of scanned compounds was narrowed down to AR only to obtain a higher sensitivity of the method.

#### 2.2.1. Sample Preparation

Liver samples were defrosted and homogenized. First, 2 g of sample was mixed with 5 mL of acetonitrile using a vortex mixer for 30 s. Then, 0.5 g of sodium acetate was added and further mixed for 1 min. The resulting suspension was sonicated for 15 min in an ultrasonic bath, mixed with a vortex mixer for 1 min, and centrifuged at 2930× *g* rcf (relative centrifugal force). After that, 0.7 mL of each supernatant were added to a new centrifuge tube with 150 mg of MgSO_4_, 50 mg of C18, and 50 mg of Primary Secondary Amine (PSA), mixed with a vortex mixer for 1 min, and centrifuged for 10 min at 2930× *g* rcf. The supernatant solutions were transferred to centrifuge filters (0.2 µm), centrifuged at 9447× *g* rcf for 10 min, and transferred to autosampler vials.

#### 2.2.2. Liquid Chromatography–Mass Spectrometry Analysis of AR

Extracts from liver samples were analyzed on a Shimadzu liquid chromatography system connected to an ABSciex API 5500 Qtrap mass spectrometer. The chromatography was performed on a C8 column (75 mm × 2.1 mm × 3 μm) with a mobile phase consisting of two solutions: 5% isopropanol in ethanol and 0.5% isopropanol in 0.1% acetic acid in water. For the AR analysis, the mass spectrometer was operating in negative ionization mode and the transition reactions monitored are shown in Table 1. Detailed data for the other compounds analyzed are available in Sell et al. [29].

#### 2.2.3. Calibration Range, Recovery, and Limit of Quantification

The whole procedure for the determination of AR was validated according to the requirements of SANTE/11945/2015. The calibration curves were prepared for each batch by fortifying fresh blank turkey liver samples from 1.00 to 250 µg/kg. The correlation coefficients (R^2^) were between 0.98–1.00. The average apparent recoveries for AR were in the range of 90–110%, with average repeatability below 12% and average within-laboratory reproducibility below 15%. The limit of quantification (LOQ) was established at 1.00 µg/kg of wet liver sample for bromadiolone, brodifacoum, coumachlor, coumatetralyl, difenacoum, flocoumafen, and warfarin and 5.00 µg/kg for chlorophacinone, diphacinone, and difethialone. Detailed data for calibration ranges, recovery, and limit of quantification of the other analyzed compounds are available in Sell et al. [29].

## 3. Results

Between 2018 and 2020, 40 samples were collected from white-tailed eagles. In ten cases (#2, #4, #13, #15, #19, #20, #29, #30, #34, #36), anatomopathological lesions, observed in varying degrees of intensity, were symptomatic for blood clotting disorders and included hemorrhages, petechiae, absence of blood clots in the heart and major blood vessels, or bleeding into body cavities (Table S1). At least one AR in all analyzed liver samples were detected and two AR compounds per liver sample were found most frequently (thirty one cases). Bromadiolone was most commonly AR-detected (thirty nine cases) followed by brodifacoum (thirty eight cases), difenacoum (six cases), and flocoumafen (two cases). Chlorophacinone, coumachlor, coumatetralyl, difethialone, diphacinone, and warfarin were not detected in any analyzed samples. The mean concentration of the sum of the AR was 174.4 µg/kg (from 2.5 to 1225.0 µg/kg). In 50% of cases, the sum of AR concentration was above 100 µg/kg, and in 25% of cases it was above 200 µg/kg. Individual data, the concentrations of AR, and their sum are presented in Table 2.

**Figure 2:**
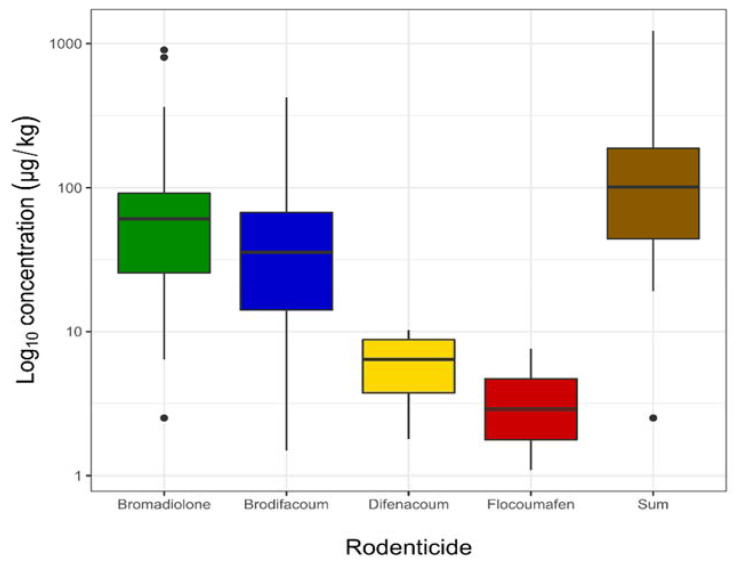
The levels of rodenticides found in white-tailed eagle (*n* = 40). The bold horizontal line on the graph represents the median; the box shows the interquartile range (25th–75th percentile); the whiskers show adjacent values (1.5 lengths of the interquartile range); dots indicate outliers.

In contrast, the levels of rodenticides are presented in Figure 2, while Table 3 shows the maximum, minimum, median, and mean concentrations in white-tailed eagle (*Haliaeetus albicilla*) liver samples.

Based on toxicological and anatomopathological investigations, AR were identified as the direct cause of death of white-tailed eagles in four cases (birds #15, #19, #30, and #34). The mean concentration of the sum of AR in these birds’ livers was 695.6 µg/kg (from 219.7 to 1225.0 µg/kg). From carbamate pesticides, in twenty-seven cases carbofuran (from 19 to 5428 µg/kg) and in five bendiocarb (from 2722 to 19487 µg/kg) were detected.

## 4. Discussion

The obtained results represent the first report about white-tailed eagles’ (*Haliaeetus albicilla*) exposure to AR in Poland. If we take into consideration different species of birds of prey, the situation is similar to other countries, concluding that the main hazards from AR are SGARs, including bromadiolone and brodifacoum [1,8,11,15,18,34]. In Finland, in addition to the SGARs, coumatetralyl (the only FGAR) was detected in 48% of the samples tested; however, its measured concentrations were low, linked to its short elimination half-life and high concentration in available products [11]. In California, AR residues were detected in thirty two out of thirty eight livers tested from birds of prey [15]. Brodifacoum was found most frequently (thirty two cases), but bromadiolone (four cases) and difethialone (two cases) were also detected [15]. The prevalence of the AR in liver samples from predatory birds species in Denmark was 91–92%, including SGARs [13]. If we consider the total concentration, SGARs accounted for 95% of the burden [13]. Our results differ from Badry et al. [35] which, as a result of the analysis of 60 samples from white-tailed eagles in Germany, detected AR only in 38.3% of samples at low concentration (from 4.69 to 28.49 µg/kg). In our study, bromadiolone and brodifacoum were detected in almost all tested liver samples, the prevalence of AR was 100%, and the concentrations of AR were significantly higher (from 1.1 to 903.0 µg/kg). The difference in results may also be due to the disparate ways samples were collected. Considering that the diet of white-tailed eagles consists mainly of fish and birds, and only occasionally mammals [30,33], dead or weakened rodents seem an unlikely source of AR for white-tailed eagles. Potential sources of AR for white-tailed eagles in Poland could be the red fox (*Vulpes vulpes*), especially during the winter period when the availability of fish is limited. The fox population in Poland is estimated at 200,000–250,000 individuals [36] and is progressively colonizing urban and suburban areas which, combined with its highly varied diet, may give it a high degree of exposure to AR. Additionally, in our experience, foxes are often targeted with bait. It was these situations in which white-tailed eagles were found near a dead fox carcass. This is usually associated with carbamates, which act quickly enough to cause the bird to fall nearby after eating the fox. Unfortunately, there is no data on the exposure of foxes to rodenticide poisoning in Poland.

Another problem we can observe in Poland is the lack of regulation and control surrounding the sale and use of particular products. For example, products containing brodifacoum and registered for professional use are freely available to anyone.

Assessing the cause of poisoning of birds of prey is often not easy, especially if we want to consider AR as a direct cause of death. Unfortunately, it is not possible to define a single threshold after which the finding would allow us to conclude AR poisoning. This is basically due to the very high variability in sensitivity to particular AR within species, races, and even within individuals or other possible situations [7]. Some authors suggest that such thresholds should be set for individual species of birds of prey, and in some publications, we can find recommended levels >100 µg/kg or >200 µg/kg as potentially lethal for birds of prey [7,21]. In our study on white-tailed eagles, we also observed signs of blood clotting disorders in many cases when the sum of AR was greater than 100 µg/kg (Table 2 and Table S1).

We would also like to draw attention to the matter that poisoning by AR should be considered more broadly beyond concentration and anatomopathological lesions. Widespread deratization influences rodent behavior, making them easy prey for predators [12], whose primary task in ecosystems is to eliminate weak and sick individuals. By looking from the other side, we should assess the not-thoroughly-investigated impact of AR on the health and behavior of birds of prey [34]. However, some areas of AR impact are challenging to define. It is difficult to determine the long-term effects on health from exposure to even low concentrations of AR over time [34]. Even a relatively small increase in blood vessel permeability can result in bruises at a much higher rate than normal. For a predator, this type of weakness may lead to searching for an easily accessible source of food, making it even more vulnerable to indirect poisoning. Often, it can be other kinds of toxic compounds, such as fast-acting carbofuran or bendiocarb, which should always be considered when studying cases of suspected AR poisoning.

As the method used in the study allowed the detection of toxic compounds from different groups, carbofuran and bendiocarb were also detected in the analyzed samples. Carbamate pesticides, both those withdrawn from use (carbofuran) and those introduced in their place (bendiocarb), are still the main cause of poisoning in Poland. There are many documented indirect poisonings by acetylcholinesterase inhibitors [27,28,37,38]. In our experience, we cannot agree with Kitowski et al. [28] that detected concentrations of carbofuran in the range 11.5–237.2 μg/kg can be classified as nonlethal. Due to its rapid metabolism, potential carbofuran poisoning should be considered in a much broader aspect than just its concentration alone in the liver, especially if we take into account the possibility of coincidence or toxic carbofuran metabolites [39].

In situations when we detect AR concentrations above 100 or 200 µg/kg combined with symptoms of blood-clotting disorders, another compound can be determined as the direct cause of death. For example, bird #20 was found dead near numerous feathers of a domestic pigeon. There was a copious mucous discharge from the beak and single drops of noncoagulated blood. The anatomopathological lesions also included noncoagulated blood flowing from the severed blood vessels and heart, numerous hemorrhages, and a small amount of bloody fluid in the abdominal cavity. In the liver sample of this white-tailed eagle, the sum of AR was 172.0 µg/kg. At first sight, it appears that the cause of death may have been AR poisoning, yet the use of the multicomponent method allowed us to detect bendiocarb at a concentration of 19,487 µg/kg. In this case, bendiocarb was found to be the direct cause of death. Another example was white-tailed eagle #2, where the use of a multicomponent method allowed identifying carbofuran as the direct cause of death (Table S1). The detection of carbofuran, due to its rapid action and metabolism, points to it as the primary cause of death. However, the hemorrhages and sum of AR detected at a level of 393.8 µg/kg pointed also to the possibility of the bird suffering an AR-induced weakness. In such cases, the detection of fast-acting and highly toxic compounds, e.g., from the carbamate group, points to them as a more probable direct cause of death. Of course, the possibility of a synergistic toxic effect of substances from both groups of compounds should also be taken into account, which would require further detailed studies.

## 5. Conclusions

White-tailed eagles had a prevalence of 100% for AR, with 50% of the eagles exceeding concentrations of 100 µg/kg in their livers. This demonstrates an extreme exposure in a top predator caused by an unregulated and intensive use of rodenticides in Poland. A likely source of exposure for white-tailed eagles may be the red fox, which is exposed to both indirect and intentional poisoning. AR products are very widely available and freely used without an appropriate level of monitoring. Specific regulations are needed in this field to limit the influence on wildlife health. Interpretation of toxicological test results in case of suspected AR poisoning should take into account not only the AR concentration and anatomopathological lesions, but also the circumstances of the death/finding of the animal followed by exclusion of other possible causes of poisoning, e.g., carbofuran, which in Poland is the leading cause of death of white-tailed eagles.

## Figures and Tables

**Table 1:** Transition reactions of analyzed AR monitored by mass spectrometer.

Analyte	Precursor Ion (Da)	Product Ions (Da)	Declustering Potential (V)	Collision Energy (CE)
Bromadiolone	525.0	181.0	−262	−47
		250.0		−47
Brodifacoum	521.1	135.1	−120	−48
		143.1		−80
Chlorophacinone	373.0	145.1	−225	−33
		201.2		−30
Coumachlor	340.9	160.9	−120	−30
		284.0		−34
Coumatetralyl	291.7	248.0	−270	−30
		142.0		−40
Difenacoum	443.0	135.0	−254	−45
		143.0		−75
Difethialone	539.1	151	−90	−50
		143		−99
Diphacinone	339.1	116.1	−254	−59
		167.2		−34
Flocoumafen	541.2	161.1	−205	−47
		382.3		−35
Warfarin	307.1	161.1	−250	−28
		250.2		−29

**Table 2:** The results of detected AR in white-tailed eagle (*Haliaeetus albicilla*) liver samples tested in years 2018–2020 in Poland.

Year	ID	Concentration (µg/kg, w.w.)				
		Bromadiolone	Brodifacoum	Difenacoum	Flocoumafen	Sum of AR
2018	#1	11.1	1.5	9.2	1.1	22.9
	#2 ^1^	358.4	35.4			393.8
	#3	79.5	14.2			93.7
	#4 ^1^	53.0	199.3			252.3
	#5		2.5			2.5
	#6	2.5				2.5
	#7	143.2	35.8			179.0
	#8	78.1	16.4			94.5
	#9	10.0	21.4			31.4
	#10	6.4	12.7			19.1
	#11	18.2	20.1	1.8		40.1
	#12	69.0	172.0			241.0
	#13 ^1^	170.0	59.6	7.6	7.6	244.8
2019	#14	19.4	61.0			80.4
	#15 ^1,2^	362.0	53.6			415.6
	#16	57.2	90.6			147.8
	#17	36.7	68.6			105.3
	#18	83.1	89.1			172.2
	#19 ^1,2^	88.2	128.2	3.3		219.7
	#20 ^1^	132.7	33.9	5.4		172.0
	#21	60.7	2.2			62.9
	#22	11.6	145.0			156.6
2020	#23	43.4	7.6			51.0
	#24	25.1	12.9			38.0
	#25	45.5	16.1			61.6
	#26	38.1				38.1
	#27	78.1	56.3			134.4
	#28	12.7	44.8			57.5
	#29 ^1^	260.0	27.1			287.1
	#30 ^1,2^	903.0	19.1			922.1
	#31	77.3	71.3			148.6
	#32	72.7	63.3			136.0
	#33	24.6	42.5			67.1
	#34 ^1,2^	802.0	423.0			1225.0
	#35	84.3	12.9			97.2
	#36 ^1^	219.0	74.3			293.3
	#37	95.4	37.5	10.2		143.1
	#38	31.5	13.6			45.1
	#39	26.0	14.1			40.1
	#40	34.0	7.6			41.6

^1^ Birds with blood-clotting disorders; ^2^ AR identified as the direct cause of death.

**Table 3:** Number of cases, maximum, minimum, median, and mean value for detected AR.

	Bromadiolone	Brodifacoum	Difenacoum	Flocoumafen	Sum of AR
Number of Cases	39/40	38/40	6/40	2/40	40/40
	Concentration (µg/kg, w.w.)				
Maximum	903.0	423.0	10.2	7.6	1225.0
Minimum	2.5	1.5	1.8	1.1	2.5
Median	60.7	35.6	6.5	4.4	101.3
Mean	121.1	58.1	6.2	4.4	174.4

## Supplementary Materials

The following supporting information can be downloaded at: https://www.mdpi.com/article/10.3390/toxics10020063/s1, Table S1: Additional information and findings from samples of white-tailed eagles (*Haliaeetus albicilla*) samples tested in years 2018–2020 in Poland in cases of suspected poisonings.
