# Supplementary material for: White-Tailed Eagles’ (Haliaeetus albicilla) Exposure to Anticoagulant Rodenticides and Causes of Poisoning in Poland (2018–2020)
Source: Toxics. 2022 Feb 1;10(2):63. doi: 10.3390/toxics10020063 (PMC8878881; doi:10.3390/toxics10020063)
Supplement: Supplementary file 1 [file toxics-10-00063-s001.zip › toxics-1540435-supplementary.pdf]

toxics

Supplementary Materials: White-tailed eagles' (*Haliaeetus albicilla*) exposure to anticoagulant rodenticides and causes of poisoning in Poland (2018–2020)

Bartosz Sell, Tomasz Śniegocki, Marta Giergiel and Andrzej Posyniak

Table S1. Additional information and findings from samples of white-tailed eagles (*Haliaeetus albicilla*) samples tested in years 2018–2020 in Poland in cases of suspected poisonings.

Year	ID	Concentration (µg/kg, w.w.)		Anatomopathological lesions / observations [a]		Age / Sex [b]	Additional findings [c]	Period of Year	Probable reason of death
		Bendiocarb	Carbofuran	Autopsy	Sampling				
2018	#1		473.1	nd	nd	nd	B	winter	carbofuran
	#2		825.8	nd	BCD, CIO	nd		winter	carbofuran
	#3		133.5	nd	nd	nd		winter	carbofuran
	#4		789.4	nd	BCD, CIO	nd		winter	carbofuran
	#5			nd	nd	nd		winter	not identified
	#6			nd	PD	nd		spring	not identified
	#7		491.6	nd	PD	nd	OS	spring	carbofuran
	#8		403.2	nd	PD	nd	OS	spring	carbofuran
	#9		238.9	nd	PD	nd	OS	spring	carbofuran
	#10			nd	nd	nd		winter	not identified
	#11		569	nd	CIO, BCO	nd		autumn	carbofuran
	#12		387	nd	CIO, BCO	nd / M	OS	autumn	carbofuran
	#13		409	nd	BCD	nd / F	OS	autumn	carbofuran
2019	#14		39.8	nd	nd	nd		winter	carbofuran
	#15			BCD, CIO		J / M		winter	anticoagulants
	#16		386	CIO, BCO		nd / F		winter	carbofuran
	#17		2707	nd	PD, BCO	nd		winter	carbofuran
	#18	5733		nd	CIO, BCO	nd		winter	bendiocarb
	#19			nd	BCD	nd		winter	anticoagulants
	#20	19487		nd	BCD, CIO	A / F	OS	spring	bendiocarb
	#21		178	nd	nd	A / nd		summer	carbofuran
	#22		222	nd	nd	nd		autumn	carbofuran
2020	#23		422	CIO		J / F		winter	carbofuran
	#24	2722		CIO		A / M		winter	bendiocarb
	#25	3579		CIO		J / F		winter	bendiocarb
	#26	17935		CIO		J / F		winter	bendiocarb
	#27			CIO, BCO		A / M		winter	not identified
	#28		137	CIO		A / M		winter	carbofuran
	#29		61.4	BCD, CIO		A / nd		winter	carbofuran
	#30			BCD		A / F		winter	anticoagulants
	#31		1404	nd	CIO	nd		winter	carbofuran
	#32		52.3	nd	PD	nd		winter	carbofuran
	#33		292	nd	nd	nd		winter	carbofuran
	#34			nd	BCD	A / F		spring	anticoagulants
	#35		19	nd	nd	nd	OS	spring	carbofuran
	#36		727	nd	BCD, CIO	nd		spring	carbofuran
	#37		122	nd	nd	nd	OS	spring	carbofuran
	#38		5211	nd	CIO	J / M		spring	carbofuran
	#39		5428	nd	CIO	J / F		spring	carbofuran
	#40		3122	nd	CIO	A / M		spring	carbofuran

[a] **BCD** – blood clotting disorders; **BCO** - blood clots were observed **CIO** - congestion of internal organs; **nd** - no data; **PD** - partially decaying;

[b] **A** – adult >3 years; **J** – juvenile < 3 years; **M** - male; **F** – female; **nd** - no data;

[c] **B** – birds founded nearby probable bait, **OS** – birds found near more dead animals of other species;
